# Progress and challenges towards targeted delivery of cancer therapeutics

**DOI:** 10.1038/s41467-018-03705-y

**Published:** 2018-04-12

**Authors:** Daniel Rosenblum, Nitin Joshi, Wei Tao, Jeffrey M. Karp, Dan Peer

**Affiliations:** 1 Laboratory of Precision NanoMedicine, School of Molecular Cell Biology and Biotechnology, George S. Wise Faculty of Life Sciences, Department of Materials Sciences and Engineering, Iby and Aladar Fleischman Faculty of Engineering; Center for Nanoscience and Nanotechnology, Cancer Biology Research Center, Tel Aviv University, Tel Aviv, 6997801 Israel; 2 Center for Nanomedicine and Division of Engineering in Medicine, Department of Medicine, Brigham and Women’s Hospital, Harvard Medical School, Cambridge, MA 02139 USA; 3 Harvard-Massachusetts Division of Health Science and Technology, Cambridge, MA 02139 USA; 4 Center for Nanomedicine and Department of Anesthesiology and, Brigham and Women’s Hospital, Harvard Medical School, Boston, MA 02115 USA

## Abstract

Targeted delivery approaches for cancer therapeutics have shown a steep rise over the past few decades. However, compared to the plethora of successful pre-clinical studies, only 15 passively targeted nanocarriers (NCs) have been approved for clinical use and none of the actively targeted NCs have advanced past clinical trials. Herein, we review the principles behind targeted delivery approaches to determine potential reasons for their limited clinical translation and success. We propose criteria and considerations that must be taken into account for the development of novel actively targeted NCs. We also highlight the possible directions for the development of successful tumor targeting strategies.

## Abstract (web summary)

Targeted delivery strategies based on nanocarriers have immense potential to change cancer care but current strategies have been shown only limited translation in the clinic. Here, the authors survey the challenge, progress and opportunities towards targeted delivery of cancer therapeutics.

## Introduction

**Fig. 1:**
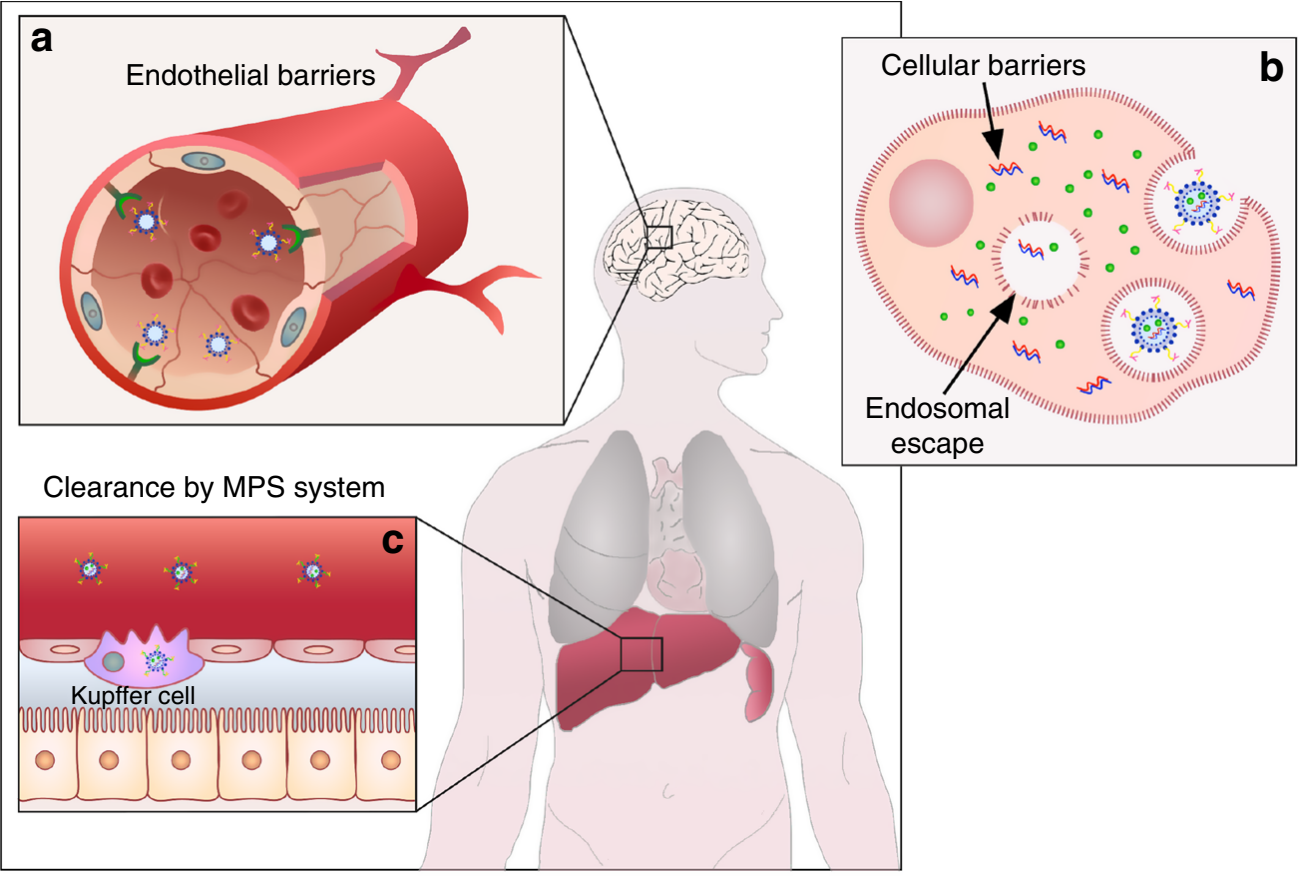
Schematic illustration of main physiological barriers faced by passive and active targeted NCs. **a** NCs face endothelial barriers in the process of their extravasation into the tumor tissue; illustration of the blood–brain barrier as an example. **b** Uptake of NCs by the target cells and their escape from the endo-lysosomal system into the cytotosl are the major cellular barriers. **c** Hepatic Kupffer cells as an example of mononuclear phagocytic system (MPS), which results in the clearence of systemically administered NCs, reducing their half-life and effective dose

Approaches for targeted delivery of therapeutics in cancer typically involves systemic administration of therapeutics packaged in nanocarriers (NCs) or localized delivery of therapeutics to the diseased tissue. Encapsulation of therapeutic molecules (e.g., small molecule inhibitors, chemotherapy, RNAi, etc.) in NCs can improve their solubility and bioavailability, alter their bio-distribution, and can also facilitate entry into the target cell. “Passively” targeted NCs, which utilize the enhanced permeability and retention (EPR) effect^1^, are the most extensively explored strategy for targeting cancer systemically. However, only a small percentage of these NCs accumulate even in high-EPR xenografted tumors (less than 1% according to a recent meta-analysis study^2^). This could be due to multiple physiological barriers (Fig. 1) and a high degree of stochasticity involved in NCs extravasation through the tumor vasculature^2^. A major proportion of NCs are also cleared by the mononuclear phagocytic system (MPS); some get physically “stuck” in the sinusoids of the liver and others are taken up by hepatocytes and Kupffer cells^3,4^.

Though multiple passively targeted NCs have been approved over the past 20 years, none of their actively targeted counterparts have advanced past clinical trials. Multiple recent reviews have summarized the current clinical status of actively targeted NCs^5–7^. Over 40,000 studies published in the last 10 years have focused on active targeting strategies and substantial progress has been made toward our understanding of how NCs interact with cells and tissues. However, we still have not been able to overcome the challenges presented by physiological barriers (Fig. 1), such as tumor penetration, tumor heterogeneity, relative hypoxia, and endosomal escape, which have limited the therapeutic benefit of actively targeted NCs. Also, the regulatory hurdles and the relatively complex scale-up of the manufacturing process of actively targeted NCs pose additional challenges toward the translation of actively targeted NCs into clinical practice.

Active cellular targeting strategies involve utilizing affinity ligands on the surface of NCs for specific homing, increased retention at the target site, and uptake by the target cells^8^. These ligands are selected to bind to overexpressed or clustered receptors on diseased tissues and cell surfaces (e.g., HER2, folate receptor, CD44, etc.)^1,8–10^. However, actively targeted NCs must first reach the target to take advantage of this increased affinity and avidity. Efficient passive targeting is therefore a prerequisite for NCs designed to systemically target tumor specific-cells or the extracellular matrix (ECM). Our understanding of EPR-related phenomena and its effect on NCs’ accumulation and penetration into tumors is mostly based on fast-growing xenografted mice models with dense vasculature, which does not recapitulate the majority of solid tumors in humans^9,11^. Several approaches have therefore been suggested to augment (e.g., TNF-α, angiotensin-II, and sonoporation) or bypass (loco-regional delivery, vasculature targeting, etc.) the EPR effect in low-EPR tumors or tumors in secluded organs (e.g., brain, bone, ovaries, bladder, etc.), respectively.

Additional obstacle toward maximizing the efficacy of NCs is pre-mature release of therapeutics. Several strategies have been devised to overcome this issue, including the development of stimuli-responsive NCs. However, these features add another level of complexity to the NC design.

In this review, we critically discuss the challenges and reasons behind limited clinical success of targeted delivery approaches in cancer treatment, in-spite of the huge number of published reports demonstrating their therapeutic potential in pre-clinical models. We also propose the focus areas for future research that will enable successful clinical translation of promising strategies for targeted delivery of cancer therapeutics.

## Crossing physiological barriers

### Passive targeting and the EPR effect

Passively targeted NCs first reached the clinic 22 years ago with the approval of PEGylated liposomal doxorubicin (DOXIL™)^12^. Passive targeting of NCs occurs due to unique characteristics of solid tumors (i.e., leaky vasculature and defective lymphatic drainage), which allow NCs to preferentially accumulate in the tumor. This phenomenon, first described in 1986 by Matsumura and Maeda, is known as the EPR effect^13,14^. A number of passively targeted NCs are currently in clinical use (e.g., Doxil™, Abraxane™, Marqibo™, DaunoXome™, and Onivyde™ in the US; Myocet™ and Mepact™ in Europe; Genexol-PM™ in Korea; and SMANCS™ in Japan)^7^. Other than that, several other NCs, including EndoTAG-1, AZD2811, and CPX-1 have also demonstrated safety and/or therapeutic effects in clinical studies^15–17^. Although most of these NCs simply alter the pharmacokinetics, toxicological profile, or solubility of drugs, few have also shown significant survival benefits and improvement in therapeutic efficacy over the parent drug in clinical studies. One example is Abraxane™ (nab-paclitaxel) that demonstrated significantly higher response rates compared to standard paclitaxel in a phase III trial conducted in patients with metastatic breast cancer^18^. Similarly, CPX-351 (Vyxeos™)—a recent FDA approved liposomal formulation of cytarabinee–daunorubicin combination has showed an improved overall survival of 9.56 months compared to 5.95 months as observed with cytarabine and daunorubicin given in their free form in patients with newly diagnosed high-risk acute myeloid leukemia^19^. This is extremely encouraging for the field and opens up new opportunities in utilizing NCs as delivery vehicles for multiple drugs.

Therapeutic efficacy of passively targeted NCs is impacted by the heterogeneity of the EPR effect within and between different tumors. Variable endothelial gaps (ranging from one to hundreds of nanometers) result in non-uniform extravasation of NCs into the tumor^20^. The tumor periphery is less permeable than the hypoxic core, which suggests that NCs should extravasate more frequently at the core than the periphery. However, multiple studies indicate the opposite—intravenously administered NCs extravasate more frequently in the tumor periphery^21,22^. Furthermore, permeability is not the only limiting factor; NC extravasation has also been shown to be governed by perfusion, which displays both spatial and temporal heterogeneity within a tumor, adding another level of complexity to controlling NC extravasation^23^. Additionally, physiochemical properties such as size and shape also affect NC extravasation and accumulation^24,25^. Even NCs that do cross the vasculature and extravasate into the tumor are impeded by the interstitial tumor matrix, which forms a barrier to their deep penetration into the tumor tissue. Reduction in the particle size significantly reduces the diffusional hindrance, thereby improving its penetration into the interstitial matrix. For example, Wong et al. proposed a multistage approach wherein a gelatin particle 100 nm in diameter upon its extravasation into the tumor tissue is reduced to a 10 nm particle via degradation by tumor-associated matrix metalloproteinases (MMPs)^26^. Similar approaches using different NCs have also been reported by many other groups^27–29^. Although promising, these approaches have not yet been evaluated clinically.

The size and geometry of NCs has also been shown to impact their other interactions with physiological barriers and tumor microenvironment. For example, Tang et al. compared the biological profiles of silica nanoconjugates with three different sizes (20, 50, and 200 nm) through both experiments and mathematical modeling^30^.The 50-nm NCs demonstrated the highest tumor penetration, most efficient uptake by tumor cells and slowest tumor clearance, resulting in highest tumor tissue retention integrated over time and highest efficacy against both primary and metastatic tumors in vivo. Similar findings correlating size of NCs to their biological effects have also been reported by multiple other groups. Other physicochemical properties of NCs, including shape, elasticity and surface charge can also impact the interaction of NCs with physiological barriers and tumor microenvironment and therefore can be crucial to optimize NC design and maximize their biological function. For example, the shape of NCs has been reported to be a crucial factor in determining their blood circulation, ability to marginate in blood vessels, and uptake by tumor cells and macrophages^31–34^. Elasticity of NCs can also impact their biological fate, including blood circulation and tumor uptake. Anselmo et al. demonstrated that softer nanoparticles (10 kPa) result in prolonged blood circulation compared to harder nanoparticles (3000 kPa) in vivo^35^. Overall, physiochemical properties of NCs can substantially impact their accumulation, retention, and penetration in tumors. However, Sykes et al. demonstrated that the optimization of physiochemical properties of NCs is specific to the target tumor’s pathophysiology, and therefore must be personalized to each tumor type and stage to maximize therapeutic efficacy^36^.

Our current understanding of EPR effectiveness is limited by the scarcity of data obtained using pre-clinical tumor models that accurately recapitulate solid tumors in humans. In fact, the most commonly used subcutaneous tumor xenografts are rapidly growing, resulting in very high-EPR tumors that could provide a false impression of the therapeutic benefit of NCs in therapies that rely on passive EPR-based targeting^11^. There is also limited patient-based experimental data on the EPR phenomenon itself and its effect on drug accumulation in the tumor site that translates into clinical efficacy^11^. Further investigation of the EPR in various human tumors and the development of better preclinical models is therefore essential for the design of NCs with better tumor penetration and therapeutic outcome^9,10^. Recently, Theek et al. investigated the correlation between tumor vascularization and EPR-based passive targeting in a subcutaneous tumor model^37^. Utilizing both contrast-enhanced functional ultrasound and computed tomography-fluorescence molecular techniques, they demonstrated heterogeneous accumulation of 10-nm near infrared-labeled polymeric NCs (pHPMA-Dy750) within and between the tumors (5%–12%). Similarly, Hansen et al. recently developed copper-64-loaded PEGylated liposomes and evaluated the EPR effect of these liposomes by microPET/CT imaging^38^. Evaluation of 11 dogs bearing spontaneous solid tumors revealed that while the EPR effect is a predominant feature in few solid tumors (e.g., carcinoma), resulting in high liposome accumulation, it could not be generalized to all solid tumors. In a more advanced study, Miller et al. showed that an FDA-approved 30-nm carboxymethyl dextran–coated magnetic nanoparticle (MNP) (ferumoxytol) could be used as a surrogate or companion particle to predict intratumoral transport, pharmacokinetics (PK), and distribution of a therapeutic NC based on poly(d,l-lactic-*co*-glycolic acid)-*b*-poly(ethylene glycol) (PLGA-PEG)^39^. Recently, Lee et al., utilized ^64^Cu-labeled HER2-targeted liposomes and PET/CT to quantify drug accumulation in 19 patients with HER2 positive metastatic breast cancer^40^. The peak liposomes accumulation was observed at 24–48 h and patients were classified based on ^64^Cu-liposomal lesion deposition using a cut-point that is comparable to a response threshold that was measured in preclinical studies. Patients with high ^64^Cu-liposomal lesion deposition were associated with more favorable treatment outcomes. These studies demonstrate that utilizing imaging techniques for evaluation and characterization of EPR could eventually enable clinicians to pre-select patients with high-EPR tumors who are likely to respond to passively targeted NCs, thus remarkably improving therapeutic outcomes.

A recent meta-analysis of pre-clinical data on NC-based delivery platforms for tumors published over the past 10 years suggested that a median of about 0.7% of the injected dose (ID) of NCs reaches the target tumors^2^. In absolute terms, this number seems small, raising serious questions about the efficiency of the EPR effect and strengthening concerns regarding treatment of low-EPR tumors. However, in relative terms, a delivery efficiency of 0.7% for NCs is substantially higher than the delivery efficiency for most of the conventional formulations of chemotherapeutics that are currently dominant in the clinic, including docetaxel, paclitaxel, and doxorubicin^41–44^. For example, a pre-clinical study by Vlerken et al. demonstrated delivery efficiency of 0.6% ID for paclitaxel-loaded NCs compared to 0.2% ID for free paclitaxel^41^. This is encouraging and clearly establishes the advantage of NCs for tumor targeted drug delivery. However, delivery efficiency of NCs can be further improved in absolute terms to maximize their therapeutic benefit. Augmenting EPR effects using angiotensin II-induced hypertension or heat-based vasodilation could be one solution, though either technique could complicate the clinical translation of NCs. Another potential and relatively translatable solution, especially for low-EPR tumors is the development of sophisticatedly engineered delivery systems that exploit non-EPR approaches for tumor targeting. For example, Xu et al. developed an injectable nanoparticle generator (iNPG) that does addresses the concerns with multiple physiological barriers^45^. Specifically, the iNPG is a discoidal micrometer-sized nanoporous silicon particle that can be loaded with drug polymer conjugates. Demonstrating tumor accumulation due to natural tropism and enhanced vascular dynamics, the iNPG releases the drug polymer conjugate, which self-assembles to form nanoparticles that are transported to the perinuclear region, thereby bypassing the drug efflux pump. iNPG showed greater efficacy in MDA-MB-231 and 4T1 mouse models of metastatic breast cancer compared to its individual components and other current therapeutic formulations. Development of such rationally engineered systems could significantly improve the delivery efficiency of NCs.

Cell-mediated delivery of NCs could be another EPR-independent approach to enhance tumor targeting in low-EPR tumors or certain metastatic tumor locations that are unreachable by passive targeting. This approach exploits the ability of certain cell types to home or migrate to such tumors^46^. Huang et al. harnessed the inherent ability of T-cells to traffic throughout the lymphatic system by conjugating nanocapsules encapsulating the topoisomerase I drug SN-38 to the cell surface^47^. Cell-mediated delivery resulted in 90-fold increase of SN-38 concentrations in lymph nodes than free drug, administered systemically at 10-fold higher doses, and also prolonged median survival by 35 days with no evidence of toxicity. In addition to targeting low-EPR tumors, immune cell-mediated delivery of NCs can also elicit improved tumor accumulation in disseminated tumors and metastases. This could open new avenues for safer targeted delivery of immunomodulating molecules such as IFN-γ, which can promote differentiation of tumor-promoting M2 macrophages to anti-tumor M1 macrophages. Furthermore, using tumor infiltrating lymphocytes or CAR T-cells for targeted delivery of NCs loaded with immunomodulating agents might enable a synergetic dual-arm treatment, augmenting anti-tumor immune responses with tumor targeting, and/or modulation of immunosuppressive cells. However, this approach is limited to therapeutics with low toxicity to normal carrier cells.

### Active targeting has great potential and greater challenges

Active cellular targeting was developed as a complementary strategy to passively targeted NCs for improving tumor localization of NCs by increasing their targeting efficiency and increasing retention at the target site^44,48,49^. However, Kirpotin et al. established that incorporation of targeting ligands on the surface of NCs increases their cellular internalization by the target cells without affecting overall tumor localization^50^. This was also confirmed by Shmidt and Wittrup, who developed a mechanistic model for better understanding and predicting the complicated relationships between molecular size, binding affinity, and tumor targeting^51^. Their model predicts that for NCs with diameters ≥50 nm active targeting does not significantly increase the tumor localization of NCs compared to non-targeted NCs. This is a major difference between actively targeted NCs compared to small molecules and antibodies.

Since targeting ligands on NCs facilitate their uptake by target cells, this strategy has been utilized to enhance the delivery of high molecular weight molecules (macromolecules, e.g., proteins, RNA, DNA, etc.) to their target cells. These molecules are sensitive to enzymatic degradation and cannot cross the cell membrane to reach their active site within target cells. However, actively targeted NCs aimed at macromolecule delivery face additional physiological barriers arising from their interaction with their target cells. One of the major barriers is escaping the endocytic pathway. After endocytosis, NCs are directed to subcellular locations by intracellular trafficking mechanisms, which may have a detrimental effect on the fate of the NCs. For example, NCs that are internalized through clathrin-mediated endocytosis (e.g., transferrin-targeted NCs) would enter the degradative pathway and eventually be degraded in lysosomes^11,13,52^. Multiple strategies have been tested to facilitate endosomal escape of NCs into the cytosol, such as pore-formation peptides and proteins, pH-buffering substances utilizing the “proton sponge effect” and fusogenic NCs^53^. However, endosomal escape of NCs in vivo remains extremely challenging. Another complexity resides in the endosomes recycling process. A study by Sahay et al. demonstrated that the vast majority (70%) of siRNA containing Lipid nanoparticles (LNPs) that were internalized underwent exocytosis^53^. Gilleron et al. recently investigated the intracellular fate of siRNA-containing LNPs in vitro and in vivo^54^. Utilizing quantitative fluorescence imaging and electron microscopy, they demonstrated that the endosomal escape of siRNA in hepatocytes occurs at very low efficiency (1–2%) and only when the LNPs reside in a specific compartment sharing early and late endosome characteristics^54^. This discovery emphasizes the importance of improving our understanding of the mechanisms of endosomal escape in different cell types to achieve improved cargo delivery efficiency, which in turn could yield better therapeutic outcomes with fewer off-target effects. Furthermore, this could have wide implications in terms of reducing the toxicity and economic concerns.

Traditionally, choosing a target for actively targeted NCs is based on “classical” or disease markers (e.g., CD19 for B-cell malignancies, HER2 for breast cancer, etc.). While these targets are clinically relevant for therapeutic monoclonal antibodies, they might not be suitable for efficient NC internalization and escape from the endosome when the therapeutics need to be delivered intracellularly (e.g., nucleic acids, intrabodies, etc.). Therefore, targets should be screened not only based on their expression levels, but also by their ability to enable rapid internalization and efficient endosomal escape. Overall, the design of actively targeted NCs as delivery vehicles is complex, since factors such as the NC’s architecture, ligand conjugation chemistry, and the choice of the ligand contribute to the efficacy of the delivery system.

Additional complexity resides in the vast heterogeneity within and between tumors and the presence of tumor and metastasis supporting stroma (e.g., tumor-associated macrophages, fibroblasts, etc.)^55^. The majority of active, cell-specific NCs target a single cell-surface receptor on tumor cells, thus disregarding tumor heterogeneity and promoting selection toward the survival of resistant clones. Consequently, current treatment usually results in apparent partial or complete responses, which in most cases followed by a resistant tumor relapse and mortality^56^. Ultimately, we argue that the development of a novel class of NCs with versatile targeting moieties or combining several NCs with different targeting moieties is crucial for improving efficacy.

Conjugation of targeting ligands to NCs results in a relatively complex manufacturing process compared to passive NCs, due to the additional steps of chemical synthesis and purification. Although this has not been the foremost limiting factor in clinical translation of actively targeted NCs, it does pose a significant challenge toward bench to bed translation of this approach due to more quality control steps, increased cost, and longer timelines. Recently, Kedmi et al., presented a possible solution to simplify the manufacturing process of actively targeted NCs and control the targeting agent orientation of the NCs’ surface^57^. In this study, they utilized a lipidated single chain Fv-based linker platform that can self-assemble into lipid-based NCs and bind IgGs from a certain isotype. The incorporation of the linker to the lipid NCs by self-assembly resolves the complexity of the chemical conjugation of antibodies to NCs as well as the need for removal of unbound antibodies. Furthermore, the use of linkers that can bind different targeting moieties can enable the development of a single versatile NC platform that can be redirected to different cellular targets simply by mixing with different targeting moieties. Such strategies might enable a real breakthrough in overcoming the challenges posed by tumor heterogeneity, without making the manufacturing process of NCs more complex.

### From protein corona to an immune barrier

**Fig. 2:**
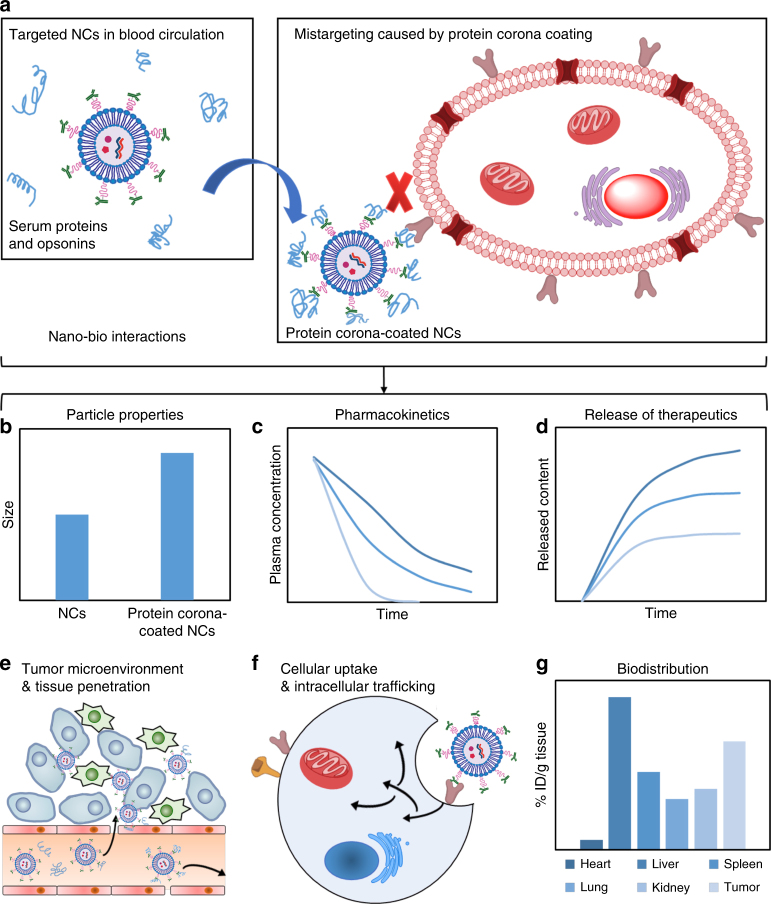
The impact of nano–bio interactions on the systemically administrated NCs. **a** During systemic circulation, targeted NCs get coated with serum proteins and opsonins, which impacts the targeting efficiency and many other properties of NCs, including **b** particle size, **c** pharmacokinetics, **d** release profiles, **e** tissue penetration, **f** cellular uptake and intrecellular trafficking and **g** biodistribution (ID injected dose)

Many factors including the route of administration and the coating formed by serum proteins and opsonins (also known as the protein corona) have been shown to affect the targeting efficiency of NCs (Fig. 2), as well as the release profiles of encapsulated therapeutics, clearance by the mononuclear phagocyte system (MPS), immunogenicity, and therapeutic efficacy^9,11,21,58^. For example, the formation of protein corona could affect many properties of NCs including size, stability and surface properties that define their cellular uptake, intracellular trafficking, pharmacokinetics, biodistribution, and toxicity^59,60^. In addition, the protein corona might mask the NC’s targeting ligands, compromising the targeting capabilities^61^. There have been numerous in vitro studies on NCs–protein interactions; however, the correlation of NCs–protein interaction in vivo with aforementioned biological responses are not yet extensively evaluated. NCs–protein interactions also restrict NCs’ circulation life due to the promotion of opsonization and recognition by MPS. However, it is worth noting that in tumors with a large volume of blood flow, NCs may not require a long circulation time to achieve effective tumor accumulation. There may also be undesirable interactions between NCs and the immune system, which in turn could lead to immunostimulation or immunosuppression. For example, the ability of some cationic NCs to activate toll-like receptor (TLR) signaling is well established^62^, but we know little about the immunological interaction of NCs with intracellular receptors such as the nucleotide-binding oligomerization domain (NOD)-like receptors (NLRs) and the inflammasome.

Various surface-coated molecules have been utilized to provide “stealth” properties to NCs during systemic circulation; the most common being PEG^63,64^. However, there is increasing evidence that the “stealthness” of PEGylated NCs is hampered by unwanted adverse effects such as complement activation, which may result in hypersensitivity reactions and even anaphylaxis^65,66^. The inadvertent recognition of NCs by the immune system may elicit a multilevel immune response, eventually leading to toxicity, adverse effects in the host and/or lack of therapeutic benefit. Thus it has become crucial to predict these reactions with the NC surface at the molecular level using appropriate pre-clinical models. This task becomes more complex in the development of actively targeted NCs. For example, when decorated with full-length IgG molecules as a targeting moiety, these NCs are recognized by the MPS via Fc receptors. This challenge could be overcome by either using smaller antibody fragments (e.g., scFv, Fab, F(ab)’_2_, etc.) or other homing molecules (e.g., aptamers, natural ligands, etc.). Functionalizing NCs with “self” markers is another strategy to limit the interactions of NCs with serum proteins and immune system during systemic circulation^67–70^.

Overall, nano–bio interaction is an extremely important research area in the development of NCs. In addition to NC–protein interaction and NC–immune system interaction during blood circulation, NC–ECM and NC–cell interaction are also crucial and have been discussed in the previous sections. With continuous improvements in our understanding of nano–bio interactions, more effective strategies could be developed to improve the targeted delivery of therapeutics to tumors.

### Active versus passive targeting

Passively targeted NCs, which rely solely on the EPR effect, may be insufficient to achieve efficient tumor targeting. We need more systematic studies to understand the interaction of NCs with physiological barriers, and the cues identified from those should be used to develop more sophisticated strategies. Utilizing natural tropism of certain cells toward specific tissues and harnessing tumor infiltrating lymphocytes are other strategies to improve NC accumulation in tumors. Recent advances in NC engineering have enabled overcoming some of the physiological barriers (e.g., ionizable lipids for endosomal escape^71–73^ and multi-layered NCs for tissue and cellular targeting^74–76^). Tools to evaluate the interaction of NCs with the immune system are already being developed (e.g., complement activation assays, cytokine induction, anti-NC antibody response, interferon response, and lymphocyte activation assays) and will enable better prediction of NC-mediated toxicity in design of future clinical trials.

Active targeting strategies are much more complex than passive approaches. In addition to the challenges associated with physiological barriers and tumor heterogeneity, a major challenge is posed by the complex design and engineering of these systems, which can complicate their pharmaceutical development and scale-up under good manufacturing practice (GMP) production and add significantly to the cost of the therapy. SynerGene Therapeutics’ SGT-94, a NC made from cationic liposomes encapsulating tumor suppressor gene RB94 and decorated with an anti-transferrin receptor (TfR) single-chain antibody fragment is an example of an actively targeted NC with a relatively simple design; it is now in a Phase I trial in patients with genitourinary tumors.

Despite multiple complexities, one major advantage of actively targeted NCs is their ability to target disseminated locations throughout the body, potentially revolutionizing the treatment of hematological malignancies (i.e., leukemia and lymphoma) and of metastatic lesions in which EPR does not play a role. However, to ensure clinical success of actively targeted NCs, significant efforts and criticism should be exercised in the design of preclinical tumor models to ensure better recapitulation of the human disease, including both solid and hematological malignancies.

Additionally, for both passive and active targeting strategies, the development of companion diagnostic imaging technologies to evaluate the targeting efficiency of NCs is crucial. Pre-selection of suitable patients and tailoring treatments to specific patients will improve tumor accumulation, treatment efficacy, and therapeutic outcomes. Furthermore, excluding unsuitable patients will reduce the incidences of adverse reactions and unnecessary treatments, reducing the expenses of both governmental health authorities and health insurance companies.

### Considerations for developing actively targeted nanocarriers

**Fig. 3:**
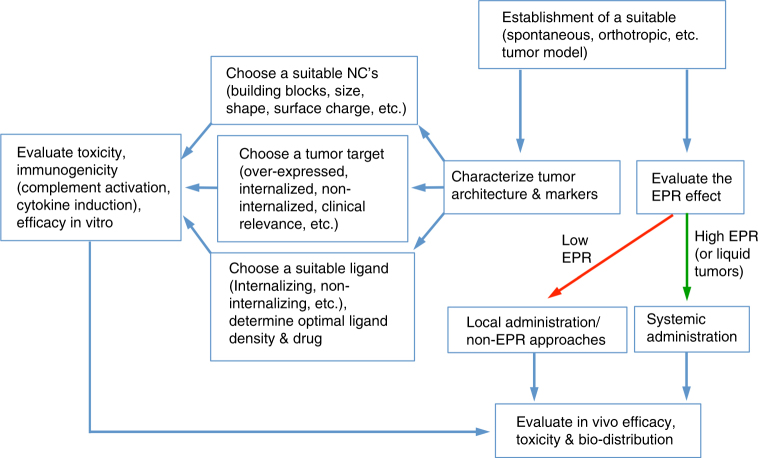
Schematic illustration of the proposed workflow in the development of actively targeted NCs

While developing actively targeted NCs, various design and biological considerations must be taken along with building a suitable experimental system to evaluate the efficacy of these systems (Fig. 3). In the case of targeted NCs intended for solid tumors, ideally it is essential to choose a model that recapitulates as much as possible the human tumor. Optimal size and physio-chemical properties should be determined by tumor architecture (density, ECM organization, etc.) and the extent of EPR effect. In addition, tools for EPR quantification should be developed in parallel to efficacy models. If the tumor site is accessible, other administration routes, for example, local delivery should be considered, thus by-passing the need of EPR effect. When choosing the target receptor and targeting moiety, several criteria should be met. In addition to choosing an exclusively or over-expressed target, several other factors must also be considered, depending on the cargo of the NCs. For example, if the active site of action for the cargo is intracellular (e.g., nucleic acids and proteins) and it cannot diffuse through the cell membrane, receptor internalization pathway is critical. In such cases, receptors that internalize via pathways that bypass the destructive endocytic pathway is preferable. Furthermore, the ability of receptor to internalize could be affected by the targeting moiety and NCs properties, such as, the binding site, receptor cluster formation, targeting moiety’s density, etc. Therefore, several targeting moieties and with varying densities for each receptor should be examined. If the NCs are intended to serve as a drug depot at the tumor site, non-internalizing and non-active strategies should be considered, thus prolonging the NCs retention at tumor site. Extra efforts should be taken in understanding the targeting moiety–receptor interaction in the context of NCs. For many years, many groups have tried to maximize the number of targeting moieties on each NC in order to exploit the avidity of multiple targeting moieties. However, there are evidences showing that this concept does not take into consideration the effects of multiple receptor binding on the target cells. Receptor clustering could activate various signaling cascades that could results in cell activation, proliferation, etc. and provoke various adverse effects. High targeting ligand density might also create a steric hindrance that could affect the receptor accessibility and increase the size of NCs. Furthermore, the strong binding by increased affinity and avidity might impact the endosomal escape of NCs upon internalization and may also affect drug release. The effect and complexity of the density of the targeting moiety was demonstrated in a recent paper by Colombo et al.^77^. In this work they used antibody-functionalized gold NCs with either single or two different types of antibodies. Although as expected, the NCs harboring two antibodies on their surface showed moderate improvement in in vitro targeting compared to NCs with single antibody, the opposite phenomenon was observed in vivo. These results highlight the concept that targeting ligand density should be optimized both in vitro and in vivo using imaging modalities and efficacy studies. To conclude, in order to exploit the advantages of actively targeted NCs, several criteria and considerations should be systematically addressed. Furthermore, additional tools, which will enable controlling the ligand density on NCs and will allow systematic study of the targeted NC–receptor interaction are lacking and must be developed.

### Controlled switch nanocarriers

**Fig. 4:**
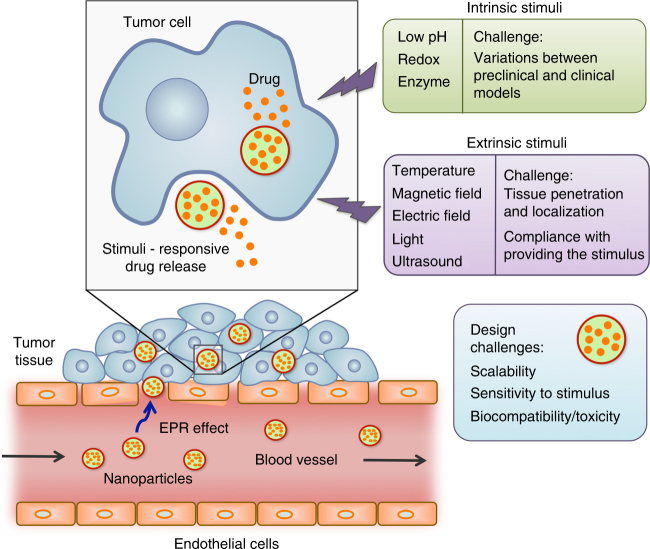
Challenges to clinical translation of stimuli-responsive NCs. Controlled-switch NCs designed to prevent premature drug release face challenges associated with the type of stimulus on which they are based. Other than that, additional design challenges for the NCs themeselves include scalability, sensitivity, and response to the stimulus, biocompatibility, and toxicity

An additional hurdle that limits the therapeutic efficacy of NCs is the premature and non-specific release of the encapsulated therapeutics. Developing stimuli-responsive NCs that can respond to specific microenvironments, releasing the therapeutics “on-demand” in a spatio-temporally controlled fashion is an attractive solution. The first report suggesting the use of stimuli-responsive NCs for cancer therapy came out in the late 1970s^78^ that described temperature-responsive liposomes for enhanced local drug release by hyperthermia. Since then, extensive studies in this arena have explored many new materials and stimuli amenable to the development of targeted and stimuli-responsive NCs in cancer therapy. Making NCs responsive to the tumor microenvironment enables spatio-temporal control of therapeutic release, offering a promising strategy to overcome the issue of premature release from NCs in systemic circulation. Designing stimuli-responsive NCs involves the use of biocompatible materials that can undergo hydrolytic cleavage, protonation, or a conformational change in response to an intrinsic or extrinsic stimulus. This approach can therefore be simple or highly complex, depending on the choice of the stimulus and the response/sensitivity of the chosen materials. Although a wide range of stimuli and a diverse library of responsive materials enable great flexibility in designing stimuli-responsive NCs, their bench-to-bed side translation is not simple. One major obstacle is the complex chemistry involved in most of these systems, which complicates their pharmaceutical development and scalability. Variations in intrinsic stimuli like pH, enzymes, and reducing agents between preclinical and clinical models also pose a challenge (Fig. 4). On the other hand, because extrinsic stimuli such as heat, light, electric fields, ultrasound, and magnetic fields can be controlled more precisely than intrinsic stimuli. Stimuli-responsive NCs that have reached clinical trials and received approval are based on extrinsic stimuli (e.g., ThermoDox, NanoTherm, and MTC-DOX)^79^. Another common attribute among these three clinically used/evaluated stimuli-responsive NCs is their simple design, which significantly facilitated their scale-up and translation. Extrinsic stimuli-based NCs hold immense potential for clinical translation in the near future; however, a few challenges still remain, including limited tissue penetration of the stimuli, poor control over their localization to prevent non-specific cell death in the surrounding tissues, and compliance with providing the stimuli (Fig. 4).

Another important concern regarding stimuli-responsive NCs is their biocompatibility; this is an issue especially when chemical synthesis is involved. Therefore, clinical translation of these systems requires extensive toxicity evaluation. Thus, a promising and translatable stimuli-responsive nanoparticle formulation should be one that involves off-the-shelf biomaterials, no major chemical modification and an extrinsic stimulus.

### External stimuli-based tumor localization

In addition to eliciting spatiotemporally controlled drug release from NCs, external stimuli, especially magnetic field, and ultrasound can also be used for tumor localization of NCs. This can be achieved by two different approaches. In one approach, the external stimuli may directly guide the NCs to the tumor site. For example, Foy et al. demonstrated that a short (1 h) exposure of a murine tumor to magnetic field resulted in enhanced accumulation of systemically administered magnetic nanoparticles (MNP) in the tumor^80^. Multiple other reports have also successfully demonstrated magnetic tumor targeting of NCs in a number of models^81,82^. Another approach to achieve external stimuli-mediated tumor localization of NCs involves the use of the stimulus to disrupt the physiological barrier, for example, the endothelium barrier, facilitating the extravasation of NCs into the target tissue. Watson et al. demonstrated the utility of ultrasound to enhance liposome accumulation in tumors by reducing their intratumoral pressure and increasing their vascular permeability^83^. Similarly, Aryal et al. utilized ultrasound to temporarily permeabilize the blood–brain barrier (BBB) and the blood–tumor barrier^84^. With multiple sessions of focused ultrasound they demonstrated improved therapeutic effect of liposomal doxorubicin in rat glioma model.

In another interesting approach, external stimuli mediated tumor targeting can be synergistically combined with active targeting, resulting in enhanced accumulation of NCs in the tumor tissue and increased therapeutic efficacy. For example, Schleich et al. developed RGD grafted PLGA-based nanoparticles loaded with paclitaxel and superparamagnetic iron oxides for dual targeting of tumor as a result of magnetic targeting and active targeting^85^. Compared to non-targeted or single targeted nanoparticles, the combination of active targeting and magnetic targeting strategies resulted in increased nanoparticle accumulation in the tumor tissue and improved therapeutic efficacy. Similar findings have been reported by Cui et al. who developed a dual-targeting strategy based on PLGA nanoparticles combining the magnetic field guidance and TfR binding^42^.

Although promising, the approach of combining external stimuli mediated targeting and active targeting may involve complex design, which may impact translatability. Therefore, careful consideration must be given to the design criteria of NCs aimed toward such dual targeting approaches. Additional challenges include limited tissue penetration of the external stimuli and compliance with providing the stimuli.

### Bypassing physiological barriers via local delivery

**Fig. 5:**
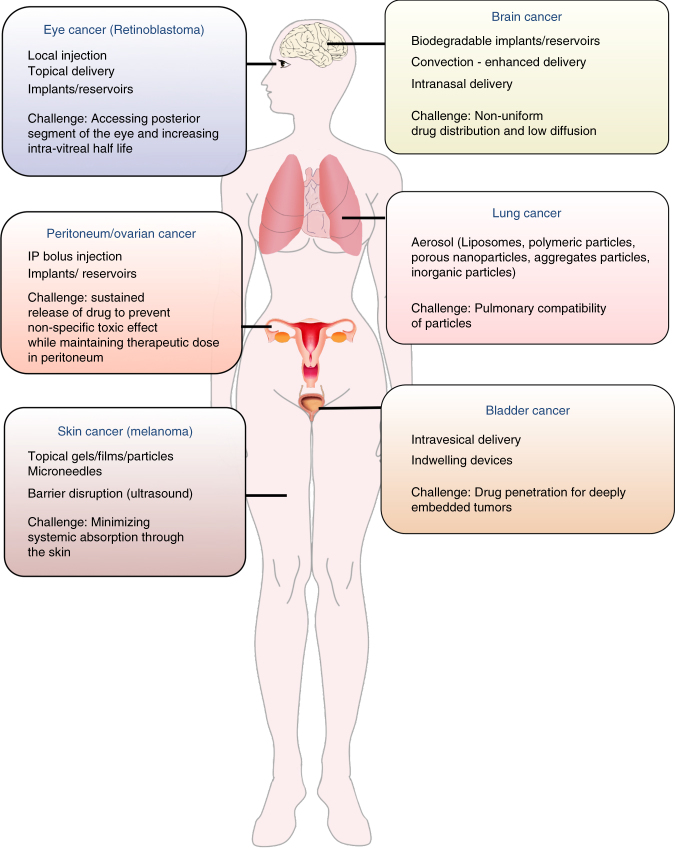
Biological and technical barriers to the success of local drug delivery. Local administration is a promising strategy for targeted drug delivery in certain cancers. However, there are still some cancer-specific biological and technical barriers that need to be overcome by the clinical success of this approach

Despite their immense potential, multiple physiological barriers must be overcome by passively and actively targeted NCs to achieve improved efficacy in clinic. Since bypassing systemic administration can help overcome such barriers, local delivery of the therapeutics directly into the diseased compartment is an attractive strategy. Certain compartments in the body including lung, bladder, brain, peritoneum, and eye can be considered unique as they can be accessed locally for the administration of therapeutics (Fig. 5). Local delivery can significantly improve pharmacological benefit at the disease site and reduce systemic toxicity compared to systemic administration. Various carriers such as liposomes, microparticles, polymeric films, and hydrogels have been developed for localized delivery to increase local residence time of the therapeutics and enable controlled release. However, although in vitro and preclinical proofs of concept have been reported for a number of NCs for local delivery in cancers, very few have reached the clinical stage. To the best of our knowledge, the only clinically approved platform for local delivery of chemotherapeutics is Gliadel®, a poly(carboxyphenoxypropane/sebacic acid) (PCPP:SA) anhydride wafer containing 3.85% biodegradable carmustine (BCNU) to treat malignant gliomas^86^. These wafers are placed along the surface of the resection cavity during surgery and can sustain release of drug for 3 weeks. However, poor penetration of the drug through the interstitial matrix and the invasiveness of the tumor cells that spread around the brain limit the efficacy of these wafers. To resolve this issue, Sawyer et al. combined polymeric controlled release with convection-enhanced delivery (CED) and developed camptothecin (CPT)-loaded poly (lactic-co-glycolic acid) (PLGA) nanoparticles that are guided via CED to a stereotactically defined location in the brain. This allows simultaneous control of location, diffusion, and duration of drug release^86^.

Local delivery of therapeutics in tumors can be further improved by developing hybrid approaches combining local drug delivery with other approaches, including active-targeting strategy and stimulus triggered drug release. Recently, Cohen et al. utilized a hybrid approach combining active-targeting strategy with local drug delivery^87^. Actively targeted hyaluronan (HA)-coated lipid nanoparticles (HA-LNPs) were administered locally to the brain in a U87MG orthotopic glioblastoma mouse model. These HA-LNPs encapsulating siRNA against Pololike kinase 1 (a G2/M transition regulator) demonstrated 80% gene knockdown in vivo and prolonged survival (≥60%) compared to control mice. In another example, Liu et al. developed implantable nanofibers for localized, near infrared (NIR) triggered delivery of doxorubicin^42^. These studies demonstrate the strong advantage of combining local delivery with other approaches, thus bypassing formerly intractable physiological barriers while augmenting therapeutic internalization and release within the target cells.

Even though this approach may be limited to certain specific cancers, it has immense potential, especially for localized chemotherapy of primary tumors that have not metastasized and when surgical resection is contraindicated. Also, for debulking surgeries that require either adjuvant or neoadjuvant chemotherapy to minimize loco-regional recurrence, localized delivery of chemotherapeutics may result in better therapeutic outcomes with reduced toxicity compared to systemic chemotherapy. For example, intraperitoneal chemotherapy before or after debulking surgery in ovarian cancer has shown therapeutic outcomes superior to systemic therapy^88^. Use of NCs in such cases can further improve efficacy due to prolonged local residence time of the therapeutics and their controlled release. As an example, tumor bed implantation of paclitaxel eluting polymeric films composed of poly(glycerol monostearate-*co*-caprolactone), following complete surgical resection showed superior efficacy compared with intravenous paclitaxel in preventing loco-regional recurrence, improving overall survival in murine recurrent sarcoma and non-small cell lung cancer models^89,90^.

Clinical success of localized therapeutic delivery approaches in cancer treatment requires addressing multiple challenges including physiological and technological barriers that are specific to the type of cancer, as indicated in Fig. 5. In addition, one major limitation of localized therapeutic delivery approaches in the context of cancer is the inability to target metastases or disseminated tumors; therefore, combining local delivery of therapeutics with other delivery strategies could provide a better package with improved outcomes.

### Regulatory and industry barriers

In addition to the challenges discussed above, obstacles related to commercialization and approval of NCs by the regulatory authorities are to date the most prominent hurdles that need to be overcome to bridge the bench-bed gap. One such obstacle is the rarity of good laboratory practices (GLP) conditions and questions regarding the validity and reproducibility of scientific results in the academic setting is a barrier to their collaboration with the pharmaceutical industry. GLP guidelines were established in response to the discovery of problems with quality and data integrity in several toxicology studies in the 1970s^91^. The FDA and later on the European Medicines Agency (EMA) adopted regulations dictating minimal standards for conducting pre-clinical laboratory studies, aimed at the commercialization of therapeutics regulated by these regulatory authorities. We argue that adopting GLP in academic pre-clinical studies is crucial to promote academia-industry collaborations, which will help in the clinical translation of academically discovered therapeutics in general, and in particular NCs. However, it is also important to realize that not all academic pre-clinical studies necessarily require GLP. For example, incorporating GLP is not crucial and may be too labor intensive for preclinical studies that aim to establish proof of concept for a new biological mechanism or technology. In some cases, it might even slow down the translation. On the other hand, GLP is truly critical when demonstrating the promise of the technology in comprehensive functional pre-clinical models with the aim of translation. Since GLP adoption can significantly increase the overall costs of a preclinical study, careful consideration of the aims of the study may help determine whether GLP is required or not. We also argue that funding organizations should allocate funds specifically for GLP, especially for proposals that have already demonstrated proof of concept and are aimed at translation.

Due to structural and chemical complexity of NCs, classical regulatory tools may not be appropriate for evaluating their safety and efficacy. Also, the need for placebo-controlled treatment regimens may need to be reconsidered. The FDA has already initiated the Nanotechnology Regulatory Science Research Plan, to address major scientific gaps in knowledge, methods, and tools required to make regulatory assessments of NCs and other nanomaterials^92^. This initiative defined five major criteria to be addressed: physio-chemical characterization, preclinical models, risk characterization, risk assessment, and risk communication. A direct example of the fruits of this initiative is the establishment of the Nanotechnology Characterization Lab (NCL), which performs thorough evaluations of nanoparticles received from academia, government, and industry. A standardized analytical cascade is employed to test physiochemical properties, pre-clinical toxicology, pharmacology, and efficacy both in vitro and in vivo (Table 1). This process enables academic laboratories and companies to gather all the needed data for filing an Investigational New Drug (IND) application to the FDA within one year. Both academia and industry must utilize such resource funding platforms for clinical translation of their NC-based therapeutics.

**Table 1:** Bridging the bench-bed gap. Summary of the key pre-clinical characterizations included in the standardized analytical cascade set by the Nanotechnology Characterization Lab (NCL) to guide both academic and non-academic research labs to gather required data for filing an investigational new drug application to the FDA

1. Physico-chemical characterization	2. In Vitro Characterization	3. In Vivo Characterization	4. Design of clinical trial and patient pre-selection
Physical properties: size, density, surface area, porosity, etc.	LAL Assays	Efficacy evaluation: therapeutics, imaging	Evaluation of the extent of EPR: MRI, PET imaging, etc. before and during clinical trials.
Surface characterization: charge, hydrophilicity, surface chemistry, solubility, etc.	Targeting efficiency: cell binding and internalization	Disposition: Tissue distribution, clearance, half-life, exposure, etc.	Target receptor profiling: MRI, PET imaging, immune staining of tumor biopsies, etc.
Stability assessment	Drug release	Single-and repeated-dose toxicity	
Batch-to-batch reproducibility: purity, sterility, uniformity, etc.	Immunological evaluation: hemolysis, platelet aggregation, plasma corona, complement activation, phagocytosis, cytokine release, etc.	Immunotoxicity	
	Toxicity: oxidative stress, cytotoxicity, etc.		
	Efficacy evaluation		

Consideration should also be given to the design of clinical trials, including selection of patient population for evaluating NC-based therapeutics. Recently, BIND-014 (PSMA-targeted docetaxel nanoparticle) failed to achieve the primary end-points in two different phase 2 clinical trials for treating non-small cell lung cancer and advanced cervical and head and neck cancer, respectively. We believe that this failure stresses the need for a paradigm shift in designing clinical trials with NCs for cancer therapy. Patient pre-selection based on the extent of EPR, the presence of target receptor and tumor heterogeneity, the ability of the actively targeted NC to bind to the target receptor and the need for companion diagnostics are crucial for achieving better therapeutic outcomes. A pioneering clinical study in that direction was conducted recently by Merrimack Pharmaceuticals to determine if tumor accumulation of ferumoxytol (FMX) iron nanoparticles, as determined by quantitative MRI, may predict response to nanoliposomal irinotecan (nal-IRI)^93^. The study showed that tumors which presented with high accumulation of FMX were more responsive to nal-IRI. We believe that such initiatives together with patient pre-selection will play an important role in bridging the bench-bed gap, promoting the development of highly characterized NCs with detailed safety and efficacy profiles together with more suitable clinical course. This will improve the chances of NCs to reach the clinic, making the promise of NCs a reality.

### Future outlook

So should one uses active cellular targeting approach? Well it is all depends on the pathological location of the tumor, it’s heterogeneity, and it’s biology (i.e., resistance, aggressiveness and the ability to fast metastasize). Passive targeting approach has great therapeutic potential; however, it is currently limited by the heterogeneity of the EPR effect and the physiological barriers associated with it. Development of rapid quantitative EPR-imaging technologies is therefore crucial for assessing the EPR effect. Applying such imaging technologies in humans should be enthusiastically pursued, as this could be a relatively quick source of critical information for future NC design. Furthermore, such technologies could provide clinicians with companion diagnostic tools useful in pre-selection of patients who would respond to EPR-based therapies, improving therapeutic outcomes. Actively targeted NCs can offer great advantages in treating hematological malignancies; however, in solid tumors they must rely on passive targeting for tumor accumulation, and therefore suffer from the same limitations as passively targeted NCs. Cellular barriers to successful intracellular delivery of the encapsulated therapeutics and complex design are some additional factors specific to the actively targeted NCs that limit their clinical success. To overcome these challenges, we have devised criteria and considerations that must be taken into account while developing actively targeted NCs.

The design of passively or actively-targeted NCs should also consider the deposition of a protein corona in physiological environment, and its consequences on biodistribution, targeting, release of therapeutics, therapeutic efficacy, and toxicity. New strategies to develop NCs with controllable/predictable biological identity are therefore necessary for accelerating the clinical translation of NCs. For the development of stimuli-responsive NCs to prevent premature release, other design challenges, such as the sensitivity of the NCs to the stimuli, scalability, and toxicity of the NCs, should also be carefully.

Several approaches have been suggested for bypassing the EPR effect, including strategies that utilize exogenous cell-mediated delivery of NCs to deliver therapeutics or immune-modulating payloads in tumors. Utilizing such strategies might provide real breakthroughs in the treatment of low-EPR solid tumors, hematological tumors, and metastatic lesions. Local delivery of therapeutics using NCs, implants, hydrogels, etc., can bypass physiological barriers associated with EPR, acting as an attractive strategy in the treatment of locally accessible tumors.

Finally, the development of better and more predictive pre-clinical animal models and adoption of GLP and standardization guidelines in academia are necessary to bridge the bench-bed gap. Such standardizations (that should include better size, surface and toxicity characterization) coupled with better understanding of tumor biology and identification of real biomarkers that can predict responders and non-responder in advance will most likely increase the success rate of translation of novel NCs into clinical practice. The Nanotechnology Regulatory Science Research Plan and similar programs established by the health authorities should be utilized to evaluate the efficacy and safety of NCs, thereby unleashing their true potential.

## References

[1] Peer D (2007). Nanocarriers as an emerging platform for cancer therapy. Nat. Nano.

[2] Wilhelm S (2016). Analysis of nanoparticle delivery to tumours. Nat. Rev. Mater..

[3] Sadauskas E (2007). Kupffer cells are central in the removal of nanoparticles from the organism. Part. Fibre Toxicol..

[4] Blanco E, Shen H, Ferrari M (2015). Principles of nanoparticle design for overcoming biological barriers to drug delivery. Nat. Biotech..

[5] Anselmo AC, Mitragotri S (2016). Nanoparticles in the clinic. Bioeng.Transl. Med..

[6] Kumari P, Ghosh B, Biswas S (2015). Nanocarriers for cancer-targeted drug delivery. J. Drug Target.

[7] Shi J, Kantoff PW, Wooster R, Farokhzad OC (2017). Cancer nanomedicine: progress, challenges and opportunities. Nat. Rev. Cancer.

[8] Saha RN, Vasanthakumar S, Bende G, Snehalatha M (2010). Nanoparticulate drug delivery systems for cancer chemotherapy. Mol. Membr. Biol..

[9] Alexis F, Pridgen E, Molnar LK, Farokhzad OC (2008). Factors affecting the clearance and biodistribution of polymeric nanoparticles. Mol. Pharm..

[10] Byrne JD, Betancourt T, Brannon-Peppas L (2008). Active targeting schemes for nanoparticle systems in cancer therapeutics. Adv. Drug Deliv. Rev..

[11] Bogart LK (2014). Nanoparticles for imaging, sensing, and therapeutic intervention. ACS Nano.

[12] Barenholz Y (2012). Doxil®—the first FDA-approved nano-drug: lessons learned. J. Control. Release.

[13] Rosenblum D, Peer D (2014). Omics-based nanomedicine: the future of personalized oncology. Cancer Lett..

[14] Matsumura Y, Maeda H (1986). A new concept for macromolecular therapeutics in cancer chemotherapy: mechanism of tumoritropic accumulation of proteins and the antitumor agent smancs. Cancer Res..

[15] Awada A (2014). A randomized controlled phase II trial of a novel composition of paclitaxel embedded into neutral and cationic lipids targeting tumor endothelial cells in advanced triple-negative breast cancer (TNBC). Ann. Oncol..

[16] Burris HA (2017). A phase I, open-label, first-time-in-patient dose escalation and expansion study to assess the safety, tolerability, and pharmacokinetics of nanoparticle encapsulated Aurora B kinase inhibitor AZD2811 in patients with advanced solid tumours. J. Clin. Oncol..

[17] Batist G (2008). A multicenter, phase II study of CPX-1 liposome injection in patients (pts) with advanced colorectal cancer (CRC). J. Clin. Oncol..

[18] Gradishar WJ (2005). Phase III trial of nanoparticle albumin-bound paclitaxel compared with polyethylated castor oil-based paclitaxel in women with breast cancer. J. Clin. Oncol..

[19] Lancet JE (2016). Final results of a phase III randomized trial of CPX-351 versus 7 + 3 in older patients with newly diagnosed high risk (secondary) AML. J. Clin. Oncol..

[20] Chauhan VP, Jain RK (2013). Strategies for advancing cancer nanomedicine. Nat. Mater..

[21] Yuan F (1995). Vascular permeability in a human tumor xenograft: molecular size dependence and cutoff size. Cancer Res..

[22] Lee H, Hoang B, Fonge H, Reilly RM, Allen C (2010). In vivo distribution of polymeric nanoparticles at the whole-body, tumor, and cellular levels. Pharm. Res..

[23] Ernsting MJ, Murakami M, Roy A, Li SD (2013). Factors controlling the pharmacokinetics, biodistribution and intratumoral penetration of nanoparticles. J. Control. Release.

[24] CabralH (2011). Accumulation of sub-100 nm polymeric micelles in poorly permeable tumours depends on size. Nat. Nano.

[25] Kolhar P (2013). Using shape effects to target antibody-coated nanoparticles to lung and brain endothelium. Proc. Natl Acad. Sci. USA.

[26] Wong C (2011). Multistage nanoparticle delivery system for deep penetration into tumor tissue. Proc. Natl Acad. Sci. USA.

[27] Tong R, Hemmati HD, Langer R, Kohane DS (2012). Photoswitchable nanoparticles for triggered tissue penetration and drug delivery. J. Am. Chem. Soc..

[28] Tong R, Chiang HH, Kohane DS (2013). Photoswitchable nanoparticles for in vivo cancer chemotherapy. Proc. Natl Acad. Sci. USA.

[29] Li HJ (2016). Stimuli-responsive clustered nanoparticles for improved tumor penetration and therapeutic efficacy. Proc. Natl Acad. Sci. USA.

[30] Tang L, Fan TM, Borst LB, Cheng J (2012). Synthesis and biological response of size-specific, monodisperse drug–silica nanoconjugates. ACS Nano.

[31] Toy R, Peiris PM, Ghaghada KB, Karathanasis E (2014). Shaping cancer nanomedicine: the effect of particle shape on the in vivo journey of nanoparticles. Nanomed.

[32] Smith BR (2012). Shape matters: intravital microscopy reveals surprising geometrical dependence for nanoparticles in tumor models of extravasation. Nano. Lett..

[33] Barua S (2013). Particle shape enhances specificity of antibody-displaying nanoparticles. Proc. Natl Acad. Sci. USA.

[34] Ananta JS (2010). Geometrical confinement of gadolinium-based contrast agents in nanoporous particles enhances T1 contrast. Nat. Nanotechnol..

[35] Anselmo AC (2015). Elasticity of nanoparticles influences their blood circulation, phagocytosis, endocytosis, and targeting. ACS Nano.

[36] Sykes EA (2016). Tailoring nanoparticle designs to target cancer based on tumor pathophysiology. Proc. Natl Acad. Sci. USA.

[37] Theek B (2014). Characterizing EPR-mediated passive drug targeting using contrast-enhanced functional ultrasound imaging. J. Control. Release.

[38] Hansen AE (2015). Positron emission tomography based elucidation of the enhanced permeability and retention effect in dogs with cancer using copper-64 liposomes. ACS Nano.

[39] Miller MA (2015). Predicting therapeutic nanomedicine efficacy using a companion magnetic resonance imaging nanoparticle. Sci. Transl. Med..

[40] Lee H (2017). 64Cu-MM-302 positron emission tomography quantifies variability of enhanced permeability and retention of nanoparticles in relation to treatment response in patients with metastatic breast cancer. Clin. Cancer Res..

[41] van Vlerken LE, Duan Z, Little SR, Seiden MV, Amiji MM (2008). Biodistribution and pharmacokinetic analysis of paclitaxel and ceramide administered in multifunctional polymer-blend nanoparticles in drug resistant breast cancer model. Mol. Pharm..

[42] Cui Y (2016). Dual-targeting magnetic PLGA nanoparticles for codelivery of paclitaxel and curcumin for brain tumor therapy. ACS Appl. Mater. & Interfaces.

[43] Peer D, Margalit R (2004). Tumor-targeted hyaluronan nanoliposomes increase the antitumor activity of liposomal doxorubicin in syngeneic and human xenograft mouse tumor models. Neoplasia.

[44] Shi J, Xiao Z, Kamaly N, Farokhzad OC (2011). Self-assembled targeted nanoparticles: evolution of technologies and bench to bedside translation. Acc. Chem. Res..

[45] Xu R (2016). An injectable nanoparticle generator enhances delivery of cancer therapeutics. Nat. Biotechnol..

[46] Levy O (2016). A prodrug-doped cellular Trojan Horse for the potential treatment of prostate cancer. Biomaterials.

[47] Huang B (2015). Active targeting of chemotherapy to disseminated tumors using nanoparticle-carrying T cells. Sci. Transl. Med..

[48] Bertrand N, Wu J, Xu X, Kamaly N, Farokhzad OC (2014). Cancer nanotechnology: the impact of passive and active targeting in the era of modern cancer biology. Adv. Drug. Deliv. Rev..

[49] Farokhzad OC, Langer R (2009). Impact of nanotechnology on drug delivery. ACS Nano.

[50] Kirpotin DB (2006). Antibody targeting of long-circulating lipidic nanoparticles does not increase tumor localization but does increase internalization in animal models. Cancer Res..

[51] Schmidt MM, Wittrup KD (2009). A modeling analysis of the effects of molecular size and binding affinity on tumor targeting. Mol. Cancer Ther..

[52] Sahay G, Alakhova DY, Kabanov AV (2010). Endocytosis of nanomedicines. J. Control. Release.: Off. J. Control. Release. Soc..

[53] Sahay G (2013). Efficiency of siRNA delivery by lipid nanoparticles is limited by endocytic recycling. Nat. Biotech..

[54] Gilleron J (2013). Image-based analysis of lipid nanoparticle-mediated siRNA delivery, intracellular trafficking and endosomal escape. Nat. Biotech..

[55] Meacham CE, Morrison SJ (2013). Tumour heterogeneity and cancer cell plasticity. Nature.

[56] Ryan S (2015). Target acquired: progress and promise of targeted therapeutics in the treatment of prostate cancer. Curr. Cancer Drug. Targets.

[57] Kedmi R (2018). A modular platform for targeted RNAi therapeutics. Nat. Nanotechnol.

[58] Bertrand N, Leroux JC (2012). The journey of a drug-carrier in the body: an anatomo-physiological perspective. J. Control. Release.

[59] Mahmoudi M, Bertrand N, Zope H, Farokhzad OC (2016). Emerging understanding of the protein corona at the nano-bio interfaces. Nano Today.

[60] Caracciolo G, Farokhzad OC, Mahmoudi M (2017). Biological identity of nanoparticles in vivo: clinical implications of the protein corona. Trends Biotechnol..

[61] Salvati A (2013). Transferrin-functionalized nanoparticles lose their targeting capabilities when a biomolecule corona adsorbs on the surface. Nat. Nano.

[62] Kedmi R, Ben-Arie N, Peer D (2010). The systemic toxicity of positively charged lipid nanoparticles and the role of Toll-like receptor 4 in immune activation. Biomaterials.

[63] Walkey CD, Olsen JB, Guo H, Emili A, Chan WC (2012). Nanoparticle size and surface chemistry determine serum protein adsorption and macrophage uptake. J. Am. Chem. Soc..

[64] Schöttler S (2016). Protein adsorption is required for stealth effect of poly(ethylene glycol)- and poly(phosphoester)-coated nanocarriers. Nat. Nano.

[65] Dobrovolskaia MA, McNeil SE (2007). Immunological properties of engineered nanomaterials. Nat. Nanotechnol..

[66] Szebeni J, Muggia F, Gabizon A, Barenholz Y (2011). Activation of complement by therapeutic liposomes and other lipid excipient-based therapeutic products: prediction and prevention. Adv. Drug. Deliv. Rev..

[67] Rodriguez PL (2013). Minimal “self” peptides that inhibit phagocytic clearance and enhance delivery of nanoparticles. Science.

[68] Parodi A (2013). Synthetic nanoparticles functionalized with biomimetic leukocyte membranes possess cell-like functions. Nat. Nano.

[69] Farokhzad OC (2015). Nanotechnology: platelet mimicry. Nature.

[70] Hu Q (2015). Anticancer platelet-mimicking nanovehicles. Adv. Mater..

[71] Semple SC (2010). Rational design of cationic lipids for siRNA delivery. Nat. Biotech..

[72] Tam YYC, Chen S, Cullis PR (2013). Advances in lipid nanoparticles for siRNA delivery. Pharmaceutics.

[73] Ramishetti S, Landesman-Milo D, Peer D (2016). Advances in RNAi therapeutic delivery to leukocytes using lipid nanoparticles. J. Drug Target..

[74] Mi Y (2016). Enzyme-responsive multistage vector for drug delivery to tumor tissue. Pharmacol. Res..

[75] Tasciotti E (2008). Mesoporous silicon particles as a multistage delivery system for imaging and therapeutic applications. Nat. Nano.

[76] Poon Z, Chang D, Zhao X, Hammond PT (2011). Layer-by-layer nanoparticles with a pH sheddable layer for in vivo targeting of tumor hypoxia. ACS nano.

[77] Colombo M (2016). Tumour homing and therapeutic effect of colloidal nanoparticles depend on the number of attached antibodies. Nat. Commun..

[78] Yatvin MB, Weinstein JN, Dennis WH, Blumenthal R (1978). Design of liposomes for enhanced local release of drugs by hyperthermia. Science.

[79] Mura S, Nicolas J, Couvreur P (2013). Stimuli-responsive nanocarriers for drug delivery. Nat. Mater..

[80] Foy SP (2010). Optical imaging and magnetic field targeting of magnetic nanoparticles in tumors. ACS Nano.

[81] Cole AJ (2011). Polyethylene glycol modified, cross-linked starch-coated iron oxide nanoparticles for enhanced magnetic tumor targeting. Biomaterials.

[82] Cole AJ, David AE, Wang J, Galbán CJ, Yang VC (2011). Magnetic brain tumor targeting and biodistribution of long-circulating PEG-modified, cross-linked starch-coated iron oxide nanoparticles. Biomaterials.

[83] Watson KD (2012). Ultrasound increases nanoparticle delivery by reducing intratumoral pressure and increasing transport in epithelial and epithelial–mesenchymal transition tumors. Cancer Res..

[84] Aryal M, Vykhodtseva N, Zhang YZ, Park J, McDannold N (2013). Multiple treatments with liposomal doxorubicin and ultrasound-induced disruption of blood–tumor and blood–brain barriers improve outcomes in a rat glioma model. J. Control. Release.

[85] Schleich N (2014). Comparison of active, passive and magnetic targeting to tumors of multifunctional paclitaxel/SPIO-loaded nanoparticles for tumor imaging and therapy. J. Control. Release.

[86] Sawyer AJ (2011). Convection-enhanced delivery of camptothecin-loaded polymer nanoparticles for treatment of intracranial tumors. Drug Deliv. Transl. Res..

[87] Cohen ZR (2015). Localized RNAi therapeutics of chemoresistant grade IV glioma using hyaluronan-grafted lipid-based nanoparticles. ACS Nano.

[88] Cristea M, Han E, Salmon L, Morgan RJ (2010). Practical considerations in ovarian cancer chemotherapy. Ther. Adv. Med. Oncol..

[89] Liu R (2012). Paclitaxel-eluting polymer film reduces locoregional recurrence and improves survival in a recurrent sarcoma model: a novel investigational therapy. Ann. Surg. Oncol..

[90] Liu R (2010). Prevention of local tumor recurrence following surgery using low-dose chemotherapeutic polymer films. Ann. Surg. Oncol..

[91] Baldeshwiler AM (2003). History of FDA good laboratory practices. Qual. Assur. J..

[92] Sanhai WR, Spiegel J, Ferrari M (2007). A critical path approach to advance nanoengineered medical products. Drug Discov. Today: Technol..

[93] Ramanathan RK (2017). Correlation between ferumoxytol uptake in tumor lesions by MRI and response to nanoliposomal irinotecan in patients with advanced solid tumors: a pilot study. Clin. Cancer Res..

